# Supplementary material for: Identification of photoperiod-induced specific miRNAs in the adrenal glands of Sunite sheep (Ovis aries)
Source: Front Vet Sci. 2022 Jul 22;9:888207. doi: 10.3389/fvets.2022.888207 (PMC9354845; doi:10.3389/fvets.2022.888207)
Supplement: Supplementary file 1 [file Data_Sheet_1.ZIP › Supplementary file/miRDeep2 file.zip!/miRDeep2 file/Know_Structure/LP42A1/oar-mir-127.pdf]

miRBase precursor : oar-mir-127
Total read count : 91382
oar-miR-127 read count : 89394
remaining reads : 1988

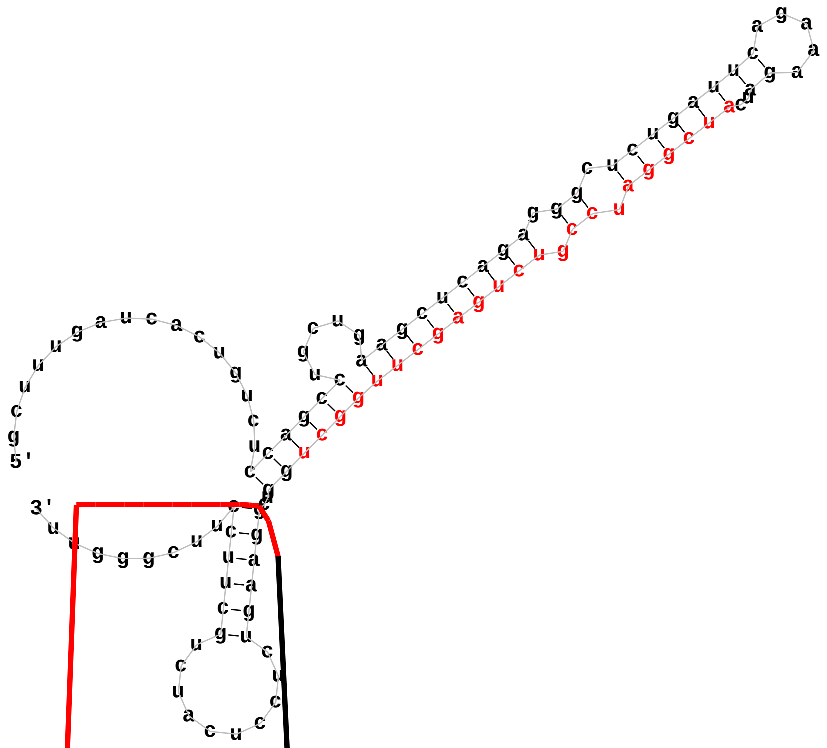

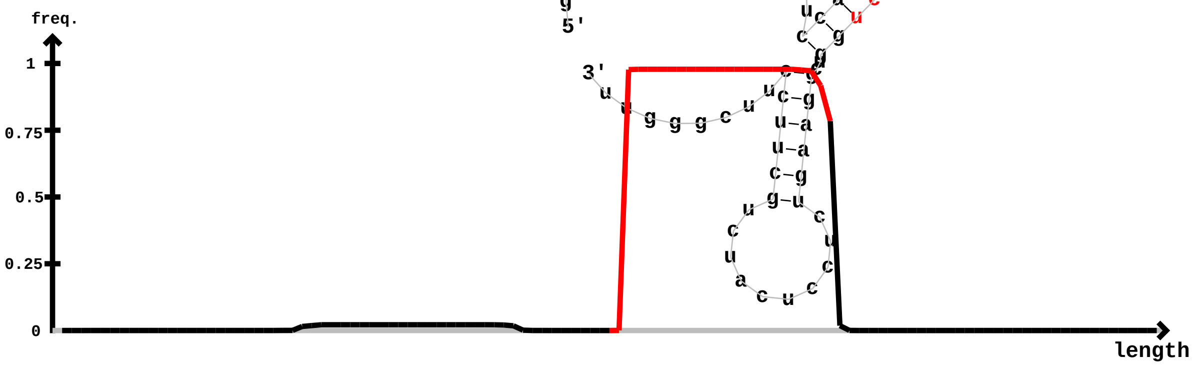

oar-miR-127

```
5'- gcuuugaucacugucuccagccugcugaagcucagagggcucugauucagaaagaucaucggauccgucugagcuuggcuggucggaagucuccucaucugcuuccuucggguu   -3'   exp
    ................((((((.....(((((((((.((.((((((((.....))..)))))).)).)))))))))))))))..((((((..........))))))........   reads   mm   sample
    .cuuugaucacugucuccagccug..........................................................................................   3       0    seq
    ..uuugaucacugucuccagccug..........................................................................................   3       0    seq
    ...uugaucacugucuccagccug..........................................................................................   1       0    seq
    ....ugaucacugucuccagccu...........................................................................................   1       0    seq
    ....ugaucacugucuccagccug..........................................................................................   3       0    seq
    .....gaucacugucuccagccug..........................................................................................   1       0    seq
    ......................ugcugaagcucagagggcucug......................................................................   7       0    seq
    ......................ugcugaagcucagagggcucuga.....................................................................   9       0    seq
    ......................ugcugaagcucagagggcucugau....................................................................   2       0    seq
    ......................ugcugaagcucagagggcucugauu...................................................................   5       0    seq
    .......................gcugaagcucagagggcucug......................................................................   3       0    seq
    .......................gcugaagcucagagggcucuga.....................................................................   2       0    seq
    .......................gcugaagcucagagggcucugau....................................................................   2       0    seq
    .......................gcugaagcucagagggcucugauu...................................................................   4       0    seq
    ........................cugaagcucagagggcucu.......................................................................   3       0    seq
    ........................cugaagcucagagggcucug......................................................................   7       0    seq
    ........................cugaagcucagagggcucuga.....................................................................   60      0    seq
    ........................cugaagcucagagggcucugau....................................................................   249     0    seq
    ........................cugaagcucagagggcucugauu...................................................................   1030    0    seq
    ........................cugaagcucagagggcucugauuc..................................................................   10      0    seq
    .........................ugaagcucagagggcucuga.....................................................................   2       0    seq
    .........................ugaagcucagagggcucugau....................................................................   13      0    seq
    .........................ugaagcucagagggcucugauu...................................................................   273     0    seq
    .........................ugaagcucagagggcucugauuc..................................................................   6       0    seq
    .........................ugaagcucagagggcucugauuca.................................................................   1       0    seq
    ..........................gaagcucagagggcucugau....................................................................   4       0    seq
    ..........................gaagcucagagggcucugauu...................................................................   174     0    seq
    ..........................gaagcucagagggcucugauuc..................................................................   96      0    seq
    ..........................gaagcucagagggcucugauuca.................................................................   5       0    seq
    ...........................aagcucagagggcucugauu...................................................................   1       0    seq
    ...........................aagcucagagggcucugauuc..................................................................   1       0    seq
    ...........................aagcucagagggcucugauuca.................................................................   1       0    seq
    ...........................aagcucagagggcucugauucag................................................................   3       0    seq
    ...........................aagcucagagggcucugauucaga...............................................................   1       0    seq
    ................................................agaaagaucaucggauccgucugagcuuggcu..................................   1       0    seq
```

oar-miR-127

```
gcuuugaucacugucuccagccugcugaagcucagagggcucugauucagaaagaucaucggauccgucugagcuuggcuggucggaagucuccucaucugcuuccuucggguu
.................................................gaaagaucaucggauccgucugagcuuggcu..................................    1       0       seq
.........................................................aucggauccgucugagcuu......................................    1       0       seq
.........................................................aucggauccgucugagcuugg....................................    14      0       seq
.........................................................aucggauccgucugagcuuggc...................................    27      0       seq
.........................................................aucggauccgucugagcuuggcu..................................    3       0       seq
..........................................................ucggauccgucugagcu.......................................    9       0       seq
..........................................................ucggauccgucugagcuu......................................    238     0       seq
..........................................................ucggauccgucugagcuug.....................................    292     0       seq
..........................................................ucggauccgucugagcuugg....................................    5035    0       seq
..........................................................ucggauccgucugagcuuggc...................................    12129   0       seq
..........................................................ucggauccgucugagcuuggcu..................................    69867   0       seq
..........................................................ucggauccgucugagcuuggcug.................................    1633    0       seq
..........................................................ucggauccgucugagcuuggcugg................................    26      0       seq
..........................................................ucggauccgucugagcuuggcuggu...............................    2       0       seq
...........................................................cggauccgucugagcuugg....................................    5       0       seq
...........................................................cggauccgucugagcuuggc...................................    19      0       seq
...........................................................cggauccgucugagcuuggcu..................................    78      0       seq
...........................................................cggauccgucugagcuuggcug.................................    9       0       seq
............................................................ggauccgucugagcuuggc...................................    1       0       seq
............................................................ggauccgucugagcuuggcu..................................    4       0       seq
............................................................ggauccgucugagcuuggcugg................................    1       0       seq
............................................................ggauccgucugagcuuggcuggu...............................    1       0       seq
```
